# *In vitro* antioxidant capacity and free radical scavenging evaluation of active metabolite constituents of *Newbouldia laevis* ethanolic leaf extract

**DOI:** 10.1186/s40659-015-0007-x

**Published:** 2015-03-14

**Authors:** Josiah Bitrus Habu, Bartholomew Okechukwu Ibeh

**Affiliations:** Bioresources Development Centre Odi, Bayelsa, National Biotechnology Development Agency, Abuja, Nigeria; Department of Biochemistry, College of Natural and Applied Sciences, Michael Okpara University of Agriculture Umudike, Umudike, Nigeria; National Biotechnology Development Agency, Abuja, Nigeria

**Keywords:** *Newbouldia laevis*, Phytochemicals, Ethanolic extraction, Antioxidants, Free radical scavengers, Bioactive constituents

## Abstract

**Background:**

The aim of the present study was to evaluate the *in vitro* antioxidant and free radical scavenging capacity of bioactive metabolites present in *Newbouldia laevis* leaf extract.

**Results:**

Chromatographic and spectrophotometric methods were used in the study and modified where necessary in the study. Bioactivity of the extract was determined at 10 μg/ml, 50 μg/ml, 100 μg/ml, 200 μg/ml and 400 μg/ml concentrations expressed in % inhibition. The yield of the ethanolic leaf extract of *N.laevis* was 30.3 g (9.93%). Evaluation of bioactive metabolic constituents gave high levels of ascorbic acid (515.53 ± 12 IU/100 g [25.7 mg/100 g]), vitamin E (26.46 ± 1.08 IU/100 g), saponins (6.2 ± 0.10), alkaloids (2.20 ± 0.03), cardiac glycosides(1.48 ± 0.22), amino acids and steroids (8.01 ± 0.04) measured in mg/100 g dry weight; moderate levels of vitamin A (188.28 ± 6.19 IU/100 g), tannins (0.09 ± 0.30), terpenoids (3.42 ± 0.67); low level of flavonoids (1.01 ± 0.34 mg/100 g) and absence of cyanogenic glycosides, carboxylic acids and aldehydes/ketones. The extracts percentage inhibition of DPPH, hydroxyl radical (OH^.^), superoxide anion (O_2_^.-^), iron chelating, nitric oxide radical (NO), peroxynitrite (ONOO^−^), singlet oxygen (^1^O_2_), hypochlorous acid (HOCl), lipid peroxidation (LPO) and FRAP showed a concentration-dependent antioxidant activity with no significant difference with the controls. Though, IC_50_ of the extract showed significant difference only in singlet oxygen (^1^O_2_) and iron chelating activity when compared with the controls.

**Conclusions:**

The extract is a potential source of antioxidants/free radical scavengers having important metabolites which maybe linked to its ethno-medicinal use.

## Background

The African continent has one of the richest biodiversity in the world and abounds in plants of economic and medicinal importance which when developed would reduce expenditure on global drug development while meeting patient’s health needs [1]. Current emphasis on healthy living based on antioxidant intake and the implication of oxidative stress molecules/free radicals on certain diseased condition [2] has generated renewed interest in screening for plants with high antioxidative properties. The identification and quantification of bioactive components that contribute to free radical scavenging activity and its consequent ethnopharmcological effect may provide link to specific drug discovery.

*Newbouldia laevis* is commonly known as African border tree. In Nigerian major languages it is called ‘Aduruku’ in Hausa, Ogirisi” in Igbo and Akoko in Yoruba [3]. *N. laevis* is a medium sized, sun loving, fast growing drought tolerant angiosperm which belongs to the Bignoniaceae family [4]. It grows up to a height of about 7–15 meters but is usually a shrub of 2–3 meters with many stemmed forming clumps of gnarled branches. In sub-Saharan Africa, the plant is used in the management of a variety of ailments for example, the bark is chewed and swallowed for stomach pains and diarrhoea as well as toothache [5]. In Nigeria and Ivory Coast, the stem bark decoctions are used for treatment of epilepsy and convulsions in children [6]. Similarly, Senegalese use the stem bark for the treatment of rheumatism especially painful arthritis of the knee. The plant also has medicinal therapy against ear aches, sore feet and chest pain [7]. Currently, leaf and root extracts of *N. laevis* have been shown to possess antimalaria [8,9] and antimicrobial activities [10,11]. The leaves, stem and fruits have been used for febrifuge, wound dressing and stomach ache medication [12].

No extensive report on the presence, and free radical scavenging activity of basic metabolites from the leaves of *N. laevis* has been provided. Similarly, investigations of the plant have produced conflicting reports on the content of phytochemical compounds present in the plant leaf thus provide scarce and inaccurate information. Furthermore, the antioxidative potential of the plant leaf have not been critically evaluated. The study therefore, evaluated the principal metabolites present in the ethanolic leaf extract of the plant as well as the antioxidant potential and free radical scavenging activity of the leaf extract. The extract was examined for different reactive oxygen species (ROS) scavenging activities including hydroxyl, superoxide, nitric oxide, hydrogen peroxide, peroxynitrite, singlet oxygen and hypochlorous acid, iron chelating capacity, antioxidant activity and metabolic constituents.

## Results

### Extractive yield

The yield of the ethanolic leaf extract of *N. laevis* was 30.3 g (9.93%).

### Phytochemical analysis

**Figure 1:**
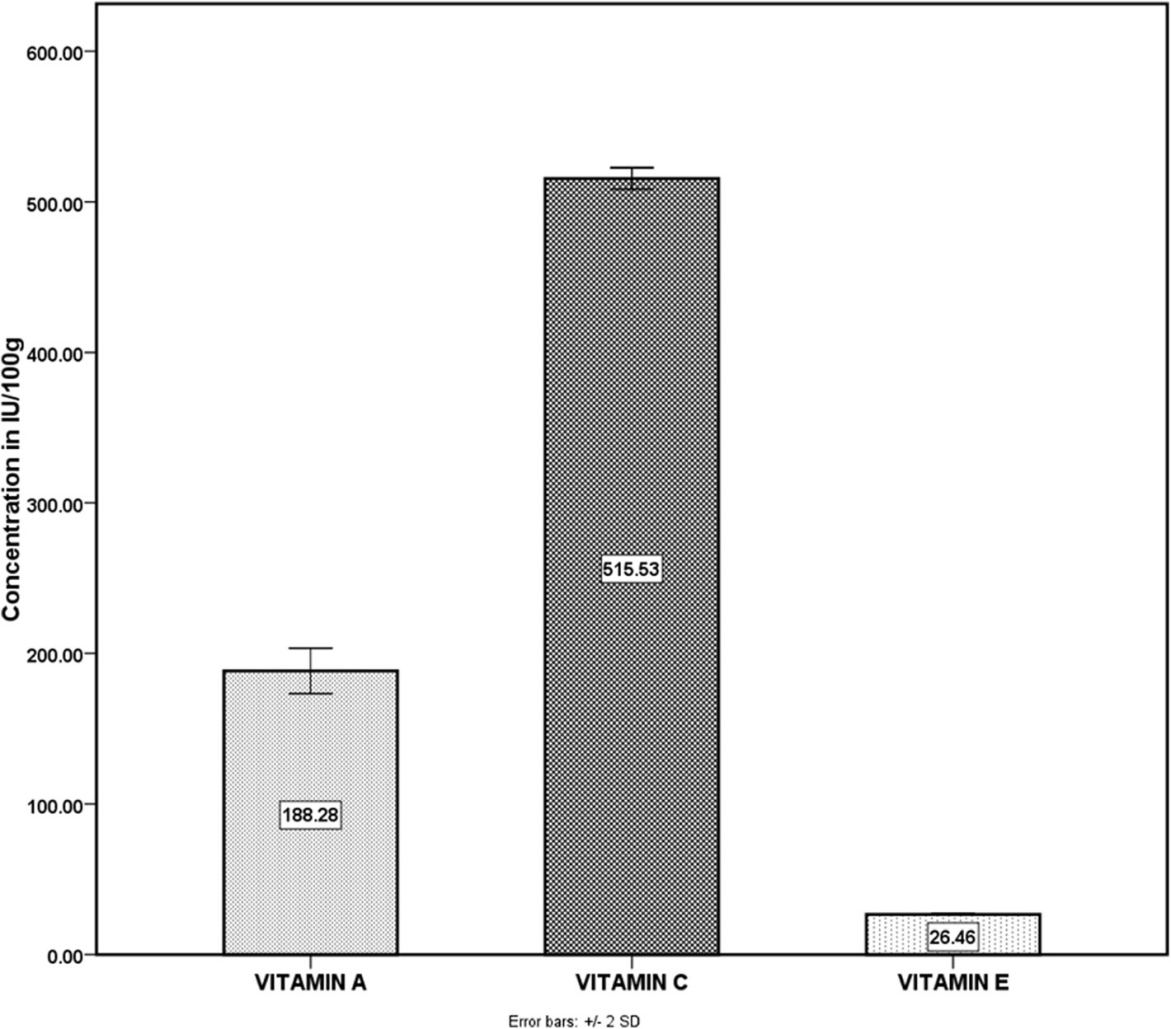
**Antioxidant vitamin composition found in the leaf extracts of** ***Newbouldia laevis*** **Data are represented as mean (n = 6).**

Preliminary phytochemical screening of *N.laevis* shows the presence of alkaloids, saponins, tannins, cardiac and steroidal glycosides, flavonoids, other metabolites were amino acids (Table 1) and vitamins A,C and E (Figure 1) while carboxylic acids, anthracene derivatives and aldehydes were absent. Evaluation of bioactive metabolic constituents gave high levels of saponins (6.2 ± 0.10), alkaloids (2.20 ± 0.03), cardiac glycosides (1.48 ± 0.22), amino acids, steroids (8.01 ± 0.04); moderate levels of tannins (0.09 ± 0.30), terpenoids (3.42 ± 0.67) and low levels of flavonoids (1.01 ± 0.34 mg/100 g) (Table 2).

**Table 1:** **Phytochemical screening of basic metabolites of the leaf extracts of** ***Newbouldia laevis***

**Plant metabolite**	**Extract content**
Cyanogenic glycosides	+
Cardiac glycosides	++
Steroid glycoside	+
Saponins	+++
Tannins	++
Alkaloids	+++
Amino acids	+++
Terpenoids	++
Flavonoids	+
Carboxylic acids	-
Aldehyde/ketones	_
Ascorbic acid	+++
Anthracene derivatives	_

+ = Trace, ++ = high, +++ = Abundant, − = Absent.

Summary of TLC phytochemical identification of *N. leavis leaf extract.*

**Table 2:** **Phytochemical composition of metabolites found in the leave extracts of** ***Newbouldia laevis (*** **mg/100 g dry weight)**

**Plant metabolite**	**Composition**
Cardiac glycosides	1.48 ± 0.22
Saponins	6.2 ± 0.10
Tannins	0.09 ± 0.30
Alkaloids	2.20 ± 0.03
Flavonoids	1.01 ± 0.34
Steroids	8.01 ± 0.04
Terpenoids	3.42 ± 0.67

Results are mean of sextuplicate determinations on a dry weight basis ± standard deviation.

### Antioxidant vitamin composition found in the leaf extracts of *Newbouldia laevis*

The result shown in Figure 1 summarizes the composition of antioxidant vitamins present in the leaves of *N. laevis* grown in Nigeria a sub-Sahara African country. The concentration of vitamins measured in IU/100 g weight shows moderate levels of Vitamin A (188.28 ± 6.19) and high levels of vitamins C (515.53 ± 12 [25.7 mg/100 g]) and E (26.46 ± 1.08).

### Antioxidant and free radical scavenging activity

The percentage inhibition of hydroxyl radical (OH^.^) and mannitol standard, superoxide anion (O_2_^.-^)/quercetin, iron chelating /EDTA, and, nitric oxide radical (NO)/curcumin, peroxynitrite (ONOO^−^)/gallic acid, singlet oxygen (^1^O_2_)/lipoic acid, hypochlorous acid (HOCl)/ascorbic acid, DPPH/ ascorbic acid, inhibition of lipid peroxidation (LPO) measured as TBARS and FRAP (Table 3) by *N. laevis* leaf extract showed a significant (P < 0.05) concentration-dependent antioxidant activity. The leaf extract had a comparable reduction capacity in all the concentrations measured when compared with the scavenging activity of known standards. The IC_50_ values of the extract showed significant difference only in singlet oxygen (^1^O_2_) (510.65 ± 9.54) vs lipoic acid standard (46.15 ± 1.16) and iron chelating (1225.05 ± 298.1) vs EDTA standard (1.27 ± 0.05) (Table 4). The FRAP of the extract at 400 μg/ml was 64 ± 2.52% (FRAP: 0.64) (Table 3) and that of the inhibition of lipid peroxidation (LPO) was 91.85 ± 0.34% (Table 3). The extract showed a good reducing power in a concentration dependent manner.

**Table 3:** **Free radical scavenging potential of** ***Newbouldia laevis*** **measured as % inhibition**

**Antioxidant activity (% Inhibition )**										
***Conc (μg/ml)***	**Hydroxyl radical (OH** ^**.**^ **)**	**Superoxide anion (O**_**2**_^**.-**^ **)**	**Iron chelating**	**Nitric oxide radical (NO)**	**Peroxynitrite (ONOO** ^**−**^ **)**	**Singlet oxygen (** ^**1**^ **O**_**2**_ **)**	**Hypochlorous acid (HOCl)**	**DPPH**	**Lipid peroxidation (LPO)**	**FRAP (mM)**
10	25.80 ± 0.03^a^	30.60 ± 0.05^a^	40.10 ± 0.02^a^	41.06 ± 0.04^a^	15.60 ± 0.01^a^	53.64 ± 0.72^a^	09.60 ± 0.12^a^	42.64 ± 1.12^a^	53.64 ± 0.82 ^a^	08.07 ± 0.31^a^
50	28.85 ± 0.01^a^	40.65 ± 0.04^b^	46.58 ± 0.04^a^	49.75 ± 0.08^b^	25.85 ± 0.04^b^	59.10 ± 0.09^a^	25.85 ± 0.31^b^	45.85 ± 0.10^a^	65.85 ± 0.09^a^ 1	18.10 ± 0.11^a^
100	50.83 ± 0.11^b^	62.74 ± 0.12^c^	65.38 ± 0.07^b^	55.13 ± 0.09^b^	35.83 ± 0.09^c^	71.62 ± 2.46^b^	35.83 ± 0.05^c^	65.85 ± 0.11^b^	85.85 ± 0.14^b^	30.15 ± 0.15^b^
200	62.97 ± 0.04^c^	69.79 ± 0.08^c^	71.67 ± 0.09^c^	61.86 ± 0.07^c^	48.97 ± 0.09^d^	86.16 ± 1.10^c^	48.97 ± 0.09^d^	79.85 ± 0.19^c^	89.85 ± 0.16^b^	50.09 ± 1.20^c^
400	76.10 ± 0.02^d^	81.11 ± 0.07^d^	90.11 ± 0.08^d^	80.08 ± 0.06^d^	62.10 ± 0.11^e^	96.00 ± 0.12^d^	62.10 ± 0.11^e^	85.85 ± 0.18^c^	91.85 ± 0.34^c^	64.01 ± 2.52^d^

Data are expressed as mean ± standard deviation (n = 6); mean in the same column with different superscripts are significantly different using Duncan’s multiple range test at p <0.05.

**Table 4:** **IC**_**50**_ **values of** ***Newbouldia laevis*** **scavenging activity and reference compounds**

**Activity**	***N. Laevis*** **IC**_**50**_	**Reference**	**IC**_**50**_
DPPH	51.4^#^	Ascorbic acid	55.4 ± 20.12**
Hydroxyl radical (OH^.^)	497.21 ± 3.65^#^	Mannitol	571.45 ± 20.12**
Nitric oxide radical (NO)	92.42 ± 2.73^#^	Curcumin	90.82 ± 4.75 (6)**
Superoxide anion (O_2_^.-^)	57.08 ± 1.22^#^	Quercetin	42.06 ± 1.35**
Peroxynitrite (ONOO^−^)	1210.83 ± 23.85^#^	Gallic acid	876.24 ± 56.96 (6)**
Singlet oxygen (^1^O_2_)	510.65 ± 9.54	Lipoic acid	46.15 ± 1.16 (6) *
Hypochlorous acid (HOCl)	276.04 ± 12.01^#^	Ascorbic acid	235.95 ± 5.75 (6)**
Iron Chelating	1225.05 ± 298.1	EDTA	1.27 ± 0.05 (6)**

Units of IC_50_ for all activities are μg/ml. Data are expressed as mean ± S.D.

EDTA = Ethylenediamine tetraacetic acid. ^#^indicates no significant difference where *p < 0.01 and **p < 0.001.

## Discussion

The investigation reported here reveals the presence of secondary metabolites such as alkaloids, tannins, flavonoids and cardiac glycosides in the ethanolic leaf extract of *N. laevis* and the free radical scavenging activity inherent in the plant species. The high antioxidant activity may relate to the plants’ curative and/or management potential of many ailments claimed in its ethno-medicine. Earlier studies on the leaf and bark extracts of the Congolese *N. leavis* showed the absence of flavonoids, saponins, quinones, terpenes and steroids [13]. Although, recent phytochemical studies on the root, bark and stem of the plant have revealed the presence of alkaloid, quinoid and phenylpropanoid compounds [14].

Phytochemical results showed the absence of carboxylic acids, aldehyde/ketones and anthracene derivatives in the ethanolic leaf extract. However, the biological active components present in the extract were vitamins A, C and E, tannins, saponins, cardiac glycosides, flavonoids, alkaloids, steroids and terpenoids, this was corroborated by previous works on phytochemicals of *N. laevis* [15]. The discordant results from several other authors [13,16] on the bioactive metabolites (especially absence of saponins) present in *N.laevis* maybe as a result of the medium of extraction (i.e. solvent), storage and environmental factors. High levels of amino acids, saponins (6.2 ± 0.10), steroids (8.01 ± 0.04), alkaloids (2.20 ± 0.03) and terpenoids (3.42 ± 0.67) characterized *N. laevis* leaf extract. The phytochemicals identified have been shown to have curative effect on several disease pathogens, thus may relate to *N. laevis* widely ethno-medicinal use [9,11]. Saponins for instance have the ability to bind sterols of cell membrane and reduce choleasterol levels hence are widely used in conventional medicines exhibiting hypocholeasterolemic effects. Generally, it could be recalled that saponins form foams in aqueous solution which have haemolytic activity and choleasterol binding properties. They have natural tendency to ward-off microbes which makes them good candidates for treating fungal and yeast infections. These compounds served as natural antibiotics that help the body to fight-off infections and microbial invasion and boost the effectiveness of certain vaccines. *N. laevis* inhibits *Staphylococcus aureus* and *Candida albicans* growth [16,17], recently the plant have been shown to also stimulate the activity of heapatic glucokinase, inhibiting glucose 6-phosphatase activity [18] thus serving as a good antidiabetic agent. The presence and concentration of these metabolites could explain the use of *N. laevis* in the treatment against various bacterial infections, sexually transmitted diseases and diabetes. The non-sugar part of saponins has a direct antioxidant activity which may contribute to the high free radical scavenging capacity of the plant leaf extract.

The trace level of cyanogenic glycoside could suggest the plant’s very low toxicity when ingested in the form of traditional medicine (Table 1). Generally, flavonoids are widely distributed group of polyphenolic compounds, characterized by a common benzopyrone ring structure that has been reported to act as antioxidants in various biological systems. The biological function of flavonoids are extended to include protection against allergies, inflammation, free radicals, platelet aggregation, microbes, ulcers, heapatoxins, viruses and tumours [19]. Germann *et. al.,* [14] revealed the presence of newbouldioside A-C and phenylethanoid glycosides in the stem bark of *N. laevis.*

Quantitative analysis of vitamins A, C and E is indicative of an enhanced free radical scavenging capacity of the plant. The leaf extract could be said to have a moderate vitamin A (188.28 ± 6.19 IU/100 g), fairly high vitamins E (26.46 ± 1.08 IU/100 g) and C (515.53 ± 12 IU/100 g) content when compared with their respective standard references. However, comparison of the vitamins showed a higher vitamin C composition. The vitamin constituents of *N. laevis* may establish in part the efficient regulation of reactive oxygen species and scavenging activity observed in the plant extract investigated in addition to maintaining membrane fluidity and integrity. Vitamin C potentially regenerates vitamin E and renews its potency. A high vitamin E content of *N. laevis* thus suffices for its antioxidant activity which is responsible for stabilization of biomembrane structure. Vitamin A on the other hand, not only contributes to the plants free radical scavenging activity but also the immunostimulatory property of *N. laevis* [20].

Phenolic compounds are very important plant constituent with multiple biological functions including antioxidant activity much related to the radical scavenging ability of their OH groups. A number of studies have reported the relative correlation between phenol and antioxidant activity [21]. It could be seen that alternative solution to synthetic drugs resides in plant natural products mostly those with free radical scavenging property. DPPH has been widely used to evaluate the antioxidant activity of natural products from plant and microbial sources. The result of the present study showed that the *in vitro* free radical potential of the extract exhibited maximum free radical scavenging activity with a comparable IC_50_ value of the known standards, except in singlet oxygen quenching and iron chelating property. The antioxidant attributes of *N. laevis* leaf extract as affected by alkaline hydrolysis and the release of bound phenolics have limited experimental evidence with few investigators reporting on stem bark [22,23]. The investigated plant metabolites with redox properties plays an important role in absorbing and neutralizing free radicals, quenching singlet and triplet oxygen, or decomposing peroxides as reported in Tables 2, 3 and 4. A higher DPPH radical-scavenging activity is associated with a lower IC_50_ value thus the results presented here indicates a higher DPPH radical–scavenging activity of the extract though not significant when compared with ascorbic acid standard (Table 4). DPPH is a stable free radical at room temperature and accepts an electron or hydrogen radical to become a stable diamagnetic molecule which is generally regarded to be a model for lipophilic radical activity. The ferric reducing power of the extract at 400 μg/ml gave 64 ± 2.52% (FRAP: 0.64) and that of inhibition of lipid peroxidation (LPO) was 91.85 ± 0.34%. The inhibition of TBARS a measure of the oxidative stress was high suggesting that *N. laevis* is a good antioxidant source. As generally observed, the antioxidant reaction of *N. laevis is* concentration-dependent which means that an increase in antioxidant activity is linearly dependent on the ethanolic leaf extract concentration of the plant (Table 3).

Hydroxyl radicals are the major active oxygen species causing lipid peroxidation and various biological damage. *N. laevis* extract was able to remove the hydroxyl radicals from the sugar component of the MDA–like oxidant and prevented the oxidative reaction. The IC_50_ value indicates that the plant extract is a better hydroxyl radical scavenger than the standard mannitol. Similarly, superoxide anion a dangerous radical to cellular components can be removed by the efficient activity of flavonoids which scavenge superoxide anions [24]. As shown in Tables 3 and 4, the superoxide radical scavenging activities of the plant extract and the reference compound quercetin are increased markedly with increasing concentrations and are comparable (no significant difference).

Nitric oxide are important in inflammatory processes but at an increased level are directly toxic to tissues resulting in vascular damage and other ailments. This toxicity is heightened on reaction with superoxide radical to form a second reactive compound peroxynitrite anion (ONOO^−^). *N. laevis* inhibits nitrite formation in the process of generating the radical (N) by direct competition with oxygen. Furthermore, the protonation of peroxynitrite (ONOO^−^) forms a dangerous and highly reactive compound peroxynitrous acid (ONOOH) [25]. The plant extract inhibits the process by scavenging peroxynitrite. *N. laevis* exhibited comparable activity with the two standards curcumin (NO) and gallic acid (ONOO^−^). HOCl inactivates catalase through breakdown of the heme prosthetic group. The plant extract inhibited catalase indicating its HOCl scavenging activity. Comparison with the ascorbic acid standard shows no significant difference. Conversely, singlet oxygen which induces hyperoxidation and oxygen cytotoxicity decreases antioxidative activity, also iron chelating effect which can stimulate lipid peroxidation are all reduced in a concentration dependent manner by the extract but not as efficient as the respective standards lipoic acid and EDTA.

## Conclusions

All extracts at tested doses (10–400 μg mL-1) revealed good scavenging activity for DPPH, FRAP, hydroxyl radical (OH^.^), nitric oxide radical (NO), superoxide anion (O_2_^.-^), peroxynitrite (ONOO^−^), singlet oxygen (^1^O_2_), hypochlorous acid (HOCl), iron chelating and inhibition of TBARS in a dose-dependent manner. The activity maybe related to the presence and concentration of secondary metabolites present in *N.laevis* leaf extract.

## Methods

### Collection and identification of plant materials

Fresh matured leaves of *N. laevis* were harvested from farms in the Department of Forestry and Environmental Management, Michael Okpara University of Agriculture, Umudike Nigeria (Latitude 05^0^ 29^1^ N to 05^0^ 42^1^, Longitude 07^0^ 24^1^ E to 07^0^ 33^1^). The matured leaves were identified and confirmed by experts of the Department of Forestry, College of Natural Resources and Environmental Management, Michael Okpara, University of Agriculture Umudike, Nigeria. A voucher specimen with the number Ibeh 2011–23 was deposited in the University herbarium for future reference.

### Sample preparation

The leaves of *N.laevis* were air-dried at room temperature and pulverized into a uniform material using a Thomas-Willey mini-milling machine (model 4, 3375-e25). Plant extraction (300 g of pulverized material) was done with 80% ethanol at 70°C by continuous percolation using Soxhlet extractor for 24 hours. The resulting extract was concentrated at 40°C in a rotary evaporator to yield a dark green mass of weight 30.3 g (9.93%). The obtained crude extract was packed ascetically in airtight plastic containers and stored at 4°C until required.

The percentage yield of the extract was calculated using the formula:

$$ \%\;\mathrm{Yield}=\frac{\mathrm{weight}\;\mathrm{of}\;\mathrm{the}\;\mathrm{extract}}{\mathrm{weight}\;\mathrm{of}\;\mathrm{plant}\ \mathrm{material}}\times \frac{100}{1}. $$

### Phytochemical determination of the metabolites

For initial phytochemical detection of major metabolites of *N. laevis* thin-layer chromatography (TLC) on silica gel 60 F_254_ with layer thickness 0.25 mm (Merck, Darmstadt, Germany) was used after dissolving the extract (2 mg) in 2 ml ethanol. The plates were developed, then left to dry for about 10 min before they were viewed under UV fluorescence light at 254 and 366 nm. Spraying was done with the required detection reagent to determine the compounds present. For flavonoids, TLC was developed in n-butanol/acetic acid/water (4:1:5), then spots were visualized with 1% AlCl_3_ solution in methanol under UV light (366 nm) (Ce 3041 Buck Scientific, UK). Alkaloids, saponins, tannins, anthraquinones, flavonoids, terpenoids, steroids and cardiac glycosides were all identified based on standard methods [26-28]. Quantitative determination was carried out by procedures previously described [29-31]. The concentration of vitamins A, E and C content of *N. laevis* was estimated using Barakat method [32] for vitamin C and Kirk and Sauya [33] for vitamins A and E.

### Assessment of inhibition of lipid peroxidation

A modified version of the thiobarbituric acid reactive substances (TBARS) assay was used to assess the extent of lipid peroxides formed using egg yolk homogenate as lipid-rich media [34]. Egg homogenate (0.5 ml, 10% in distilled water v/v) was added to 0.1 ml of extract and the volume made up to 1 ml with distilled water. A volume of 0.05 ml of 0.07 M FeSO_4_ was added to the above mixture and further incubated for 30 min, to induce lipid oxidation. Then 1.5 ml of 20% acetic acid (pH 3.5), 1.5 ml of 0.8% w/v TBA prepared in 1.1% w/v sodium duodecyl sulphate and 0.05 ml of 20% w/v TCA were sequentially added. The resulting mixture was vortexed and heated at 95°C for 60 min. After cooling, 5 ml of butan-1-ol was added and the mixture centrifuged at 3000 rpm for 10 min (Ultra-8 digital CR Scientific, Koningsweg, Netherlands). The absorbance of the organic upper layer was measured at 532 nm and converted to percentage inhibition using the formula: Varying concentrations (10 to 400 μg/ml) of the extract was used for all free radical scavenging (LPO,FRAP, ONOO^−^,HOCl,^1^O_2,_^,^NO, OH, Fe^2+^ chelation, DPPH, O_2_^.-^) analysis. Free radical scavenging potential of *N. laevis* was measured as % Inhibition and the IC_50_ values determined in each parameter, comparison were made with corresponding reference compounds. It is imperative to note that the choice of assay standards were made to effectively evaluate the scavenging property of the extract using specific known and well characterized compounds.

1 $$ \mathrm{Inhibition}\ \mathrm{of}\ \mathrm{Lipid}\ \mathrm{Peroxidation}\left(\%\right)=\left(1\hbox{--} \mathrm{E}\kern0.15em /\kern0.15em \mathrm{C}\right)\mathrm{X}100 $$

Where C = absorbance of fully oxidized control and E = absorbance in the presence of extract.

### Ferric reducing potential assay

The reductive potential (ferric reducing antioxidant power; FRAP) of *N. laevis* was determined based on the chemical reduction of Fe^3+^ to Fe^2+^ [35]. Briefly, 50 μl of the extract was added to 1.5 ml of freshly prepared and pre-warmed (37°C) FRAP reagent (300 mM acetate buffer, pH = 3.6, 10 mM tripyridyl-s-triazine (TPTZ) in 40 mM HCl and 20 mM FeCl3.6H2O in the ratio of 10:1:1) and incubated at 37°C for 10 min. The absorbance of the sample was read against reagent blank (1.5 ml FRAP reagent and 50 μl distilled water, [MI]) at 593 nm. Standard solutions of Fe2+ in the range of 100 to 1000 mM were prepared from ferrous sulphate (FeSO4.7H2O) using distilled water. Thus at low pH, the reduction of ferric tri (2-pyridyl)-1, 3, 5-triazine (Fe III TPTZ) complex to ferrous form (FRAP value) was measured by monitoring the change in absorption at 593 nm. Absorbance (A) readings were taken after 0.5 s and every 15 s thereafter during the monitoring period. The change in absorbance (ΔA_593nm_) between the final reading selected and the M1 reading was calculated for each sample and related to (ΔA_593nm_) of a Fe^II^ standard solution tested in parallel. The reaction was monitored for up to 8 min but the 4-min readings were selected for calculation of FRAP values. The final result was expressed as concentration of antioxidant having a ferric reducing ability equivalent to that of 1 mmol/L FeSO4. The calculation was done by:

2 $$ \mathrm{FRAP}\ \left(\mathrm{m}\mathrm{M}\right) = \frac{\left(\varDelta {\mathrm{A}}_{593\mathrm{nm}}\kern0.5em  of\  sample\; from\ \mathit{\mathsf{0}}\mathit{\hbox{-}}\mathit{\mathsf{4}} min\right)\kern0.5em }{\left(\varDelta {\mathrm{A}}_{593\mathrm{nm}} of\kern0.5em  standard\  from\ \mathit{\mathsf{0}}\ to\ \mathit{\mathsf{4}}\  min\right).} \times FRAP\; value\  of\; standard\kern0.24em \left(1000\ mM\right) $$

### DPPH based free radical scavenging activity

DPPH radical scavenging activity was detected for antioxidant activity by thin layer chromatography (TLC) screening through spotting a concentrated ethanolic solution of the extract on silica gel plates. The plates were developed in ethanol: ethyl acetate (2:1) then air-dried and sprayed with 0.2% w/v DPPH spray. The presence of yellow spots were detected. Radical scavenging activity of extracts was measured according to the DPPH spectrophotometric method [36] using vitamin C (Emzor Pharmaceutical Industries, Nigeria) as a reference antioxidant. Ethanol (1.0 ml) plus extract solution (2.5 ml) was used as blank while 1 ml of 0.3 mm DPPH plus ethanol (2.5 ml) was used as a negative control. The free radical scavenging properties of the extracts against 2, 2-diphenyl-1-picryl hydrazyl (DPPH) radical were measured at 518 nm, as an index of their antioxidant activity. IC_50_ values (the concentration of extracts required to scavenge 50% of DPPH free radicals) were also calculated. The absorbance (abs) of the resulting mixture measured at 518 nm was converted to percentage antioxidant activity (AA %) and thus calculated by the equation:

3 $$ \mathrm{AA}\%=\left[100\hbox{--} \left(\left(\mathrm{ABS}\,\, \mathrm{sample}\, \hbox{---}\,  \mathrm{ABSblank}\right)\times 100\right)\right]/\mathrm{ABScontrol} $$

### Superoxide radical scavenging activity

Measurement of superoxide radical scavenging capacity of *N. laevis* extracts was done using a previously reported method [37] described by Fontana *et al*. The reaction mixture (1 ml) contained phosphate buffer (20 mM, pH 7.4), NADH (73 μM), nitroblue tetrazolium (NBT) solution (50 μM), Phenazine methosulphate (PMS) solution (15 μM) and various concentration of the plant extract as described elsewhere. The PMS/NADH system generates superoxide radicals, which reduce NBT to a purple formazan. This was incubated at 25°C for 5 mins and absorbance measured at 562 nm against the ethanol blank to determine the quantity of formazan. Thus the assay of SOD is based on the inhibition of the formation of NADH-phenazine methosulphate-nitroblue tetrazolium formazan. Quercetin was used as a standard and the percentage inhibition of superoxide anion generation was calculated as previously described in equation 3.

### Nitric oxide radical scavenging assay

Ebrahimzadeh *et al*. [38] procedure was adopted to determine the scavenging activity of the plant extracts against nitric oxide radical. Nitric oxide was generated from sodium nitroprusside and measured by the Greiss reaction. Curcumin was used as a standard. Curcumin inhibits induction of nitric oxide synthase and is a naturally occurring direct scavenger of nitric oxide. It reduces the amount of nitrite formed between oxygen and nitric oxide generated from sodium nitroprusside. The absorbance was measured at 596 nm and the percentage antioxidant activity calculated using the formula in equation 3.

### Hydroxyl radical scavenging assay

The scavenging activity of the extract against hydroxyl radical was measured using the deoxyribose test-tube method [39] with minor changes. All solutions used was freshly prepared; 200 μL of 2.8 mM 2-deoxy-2-ribose, 5 μL of *N.laevis* leaf extract ,400 μL of 200 mM FeCl_3,_ 1.04 mM EDTA, 200 μL H_2_O_2_ (1.0 mM), 200 μL ascorbic acid(1.0 mM) and various concentrations (10–400 μg/ml) of the plant extract was mixed to form a reaction mixture. The mixture was incubated for 1 hour at 37°C. The extent of deoxyribose degradation was measured by TBA reaction. TCA (1.5 ml of 2.8% TCA) was added and kept for 20 mins. The solution was incubated at 90°C for 15 min to develop the colour. Afterwards, the solution was cooled and the absorbance measured at 532 nm against an appropriate blank solution Mannitol, a classical •OH scavenger was used as a positive control. The percentage antioxidant activity was calculated using the formula described in equation 3.

### Peroxynitrite scavenging

Peroxynitrite (ONOO^−^) was synthesized as described by previous methods [40]. An acidic solution (0.6 M HCl) of 5 ml H_2_O_2_ (0.7 M) was mixed with 5 ml 0.6 M KNO_2_ on an ice bath for one second and 5 ml of ice-cold 1.2 M NaOH was added. Excess H_2_O_2_ was removed by treatment with granular MnO_2_ prewashed with 1.2 M NaOH and the reaction mixture was left overnight at −20°C. Collection of peroxynitrite solution was achieved through the top of the frozen mixture and the concentration measured spectrophotometrically at 302 nm (ε = 1670 M^−1^ cm^−1^). The peroxynitrite scavenging activity was determined by Evans Blue bleaching assay [41] with slight modification. The reaction mixture contained 50 mM phosphate buffer (pH 7.4), 0.1 mM DTPA, 90 mM NaCl, 5 mM KCl, 12.5 μM Evans Blue, various concentrations of the plant extract (10–400 μg/ml) and 1 mM peroxynitrite in a final volume of 1 ml. The absorbance was measured at 611 nm after 30 min incubation at 25°C for. The percentage scavenging of ONOO^−^ was calculated by comparing the results of the test and blank samples. Gallic acid was used as the standard.

### Singlet oxygen scavenger

Production of singlet oxygen (^1^O_2_) was achieved by monitoring ***N***, ***N***-dimethyl-4-nitrosoaniline (RNO) bleaching, using a previously reported method [42,43]. Singlet oxygen was generated by a reaction between NaOCl and H_2_O_2_ and the bleaching of RNO monitored at 440 nm. The reaction mixture contained 45 mM phosphate buffer (pH 7.1), 50 mM NaOCl, 50 mM H_2_O_2_, 50 mM histidine, 10 μM RNO and various concentrations (10–400 μg/ml) of the plant extract in a final volume of 2 ml. It was incubated at 30°C for 40 min and the decrease in RNO absorbance was measured at 440 nm. The scavenging activity of sample was compared with that of lipoic acid, used as a standard compound.

### Hypochlorous acid scavenging

Pedraza-Chaverrí *et al.* [44] description of hypochlorous acid scavenging activity was adopted with minor modification to determine the hypochlorous acid scavenging activity of *N.laevis.* Hypochlorous acid (HOCl) was prepared immediately before the experiment by adjusting the pH of a 10% (v/v) solution of NaOCl to 6.2 with 0.6 M H_2_SO_4_ and the concentration of HOCl was determined by measuring the absorbance at 235 nm using the molar extinction coefficient of 100 M^−1^ cm^−1^. The scavenging activity was evaluated by determining the decrease in absorbance of catalase at 404 nm. The reaction mixture final volume (1 ml) contained 50 mM phosphate buffer (pH 6.8), catalase (7.2 μM), HOCl (8.4 mM) and increasing concentrations (10–400 μg/ml) of plant extract. The assay mixture was incubated at 25°C for 20 min and the absorbance measured against an appropriate blank. Ascorbic acid, a potent HOCl scavenger, was used as a standard.

### Chelation power on ferrous (Fe^2+^) ions

The ferrous ion chelating activity of the extract was evaluated *in vitro* as previously reported [45] with minor alterations. The reaction was carried out in HEPES buffer (20 mM, pH 7.2).Various concentrations (10–400 μg/ml) of the plant extract was added to a solution of 2 mM FeCl 2 (0.05 ml). The reaction was initiated by the addition of 5 mM ferrozine (0.2 ml) and the mixtures was then shaken vigorously and incubated at room temperature for 20 min. The absorbance of the solution was measured spectrophotometrically at 562 nm. The percentage inhibition of ferrozine-Fe^2^+ complex formation (ferrous ion chelating ability) was calculated as [(A0 –A1/As)/A0] x100, where A0 is the absorbance of the control, and A1 is the absorbance of the plant extract and As the absorbance of a standard solution. EDTA was used as a standard.

### Statistical analysis

The statistical analysis was done by one-way analysis of variance (ANOVA) using spss® version 18. The differences between the means were tested using posthoc LSD. A *p*-value of p <0.05 was considered to be statistically significant and result presented as mean ± standard deviation. All assays were done in sextuplicate. The IC_50_ values were calculated by the formula Y = 100*A1/(X + A1), where A1 = IC_50_, Y = response (Y = 100% when X = 0), X = inhibitory concentration. The IC_50_ values were compared by paired t tests and the antioxidant activity expressed in terms of IC_50_ (μg/ml concentration required to inhibit the radical formation by 50%).

### Ethical approval

All authors hereby declare that all experiments have been examined and approved by the appropriate ethics committee and have therefore been performed in accordance with the ethical standards laid down in the 1964 Declaration of Helsinki and Michael Okpara University, Umudike, Nigeria.
